# Effects of Moderate Electric Field Pretreatment on the Efficiency and Nutritional Quality of Hot Air-Dried Apple Slices

**DOI:** 10.3390/foods14132160

**Published:** 2025-06-20

**Authors:** Deryanur Kalkavan, Nese Sahin Yesilcubuk

**Affiliations:** 1 Department of Food Engineering, Chemical and Metallurgical Engineering Faculty, Istanbul Technical University, Istanbul 34469, Turkey; kalkavand@itu.edu.tr; 2 Beko Corporate Central R&D, Food&Cooking Technologies Management, Istanbul 34950, Turkey

**Keywords:** moderate electric field, pulsed electric field, MEF, PEF, waveform, drying

## Abstract

This study investigates the effects of electric field pretreatment parameters such as electric field strength (0.1–0.2 kV/cm), waveform (sinusoidal vs. square), and application mode (continuous vs. pulsed) on the quality attributes of dried Fuji apple slices, including ascorbic acid (vitamin C) retention, β-carotene content, and hydroxymethylfurfural (HMF) formation. Electric-field-treated samples were compared to untreated controls after convective drying at 75 °C. Results revealed that vitamin C was significantly influenced by waveform, with sinusoidal waves preserving about 27% more vitamin C than square waves, likely due to reduced oxidative degradation from gentler electroporation. Conversely, square waves caused the highest β-carotene losses (25% vs. control), attributed to prolonged peak voltage destabilizing carotenoids. HMF formation was reduced by 10–23% in electric-field-treated samples compared to controls, linked to accelerated drying rates limiting Maillard reaction time. Low electric field strengths (0.1–0.15 kV/cm) enhanced antioxidant activity; however, higher intensities showed a potential decline. The square waveform had a more detrimental effect on phenolic compounds than the sinusoidal waveform. These findings suggest that low electric field pretreatment, particularly with sinusoidal waveforms at 0.2 kV/cm, enhances drying efficiency while balancing nutrient retention and HMF mitigation, offering a promising strategy for producing high-quality dried fruits.

## 1. Introduction

The global demand for nutrient-rich and shelf-stable fruits has caused a shift in consumer preferences toward minimally processed and additive-free foods. Apples, a widely consumed fruit rich in vitamin content and phenolic compounds, are particularly vulnerable to quality degradation during conventional hot air drying. Prolonged drying times lead to discoloration due to the activity of oxidative enzymes and high temperatures accelerate the oxidation of heat-sensitive nutrients while promoting the formation of hydroxymethylfurfural (HMF), a Maillard reaction byproduct with potential carcinogenic properties [1]. These challenges highlight the need for innovative pretreatments that enhance drying efficiency without compromising the nutritional quality of food products.

Pulsed electric field (PEF) technology has emerged as a promising non-thermal pretreatment to modify cellular structures through electroporation, thereby accelerating moisture removal and reducing thermal exposure. However, its efficacy is highly dependent on waveform characteristics, electric field strength, and pulsing parameters. Square waveforms, characterized by rapid voltage spikes and sustained peak intensities, are known for superior energy efficiency and cell-disruption capabilities compared to sinusoidal or exponential waveforms. Yet, their aggressive electroporation may increase oxidative damage to compounds like vitamin C and carotenoids. This trade-off between nutrient retention and processing efficiency remains poorly understood, particularly in plant tissues with complex cellular matrices [2].

To date, most studies have primarily focused on the efficiency of electric field treatments on microbial inactivation or juice extraction, with limited attention to its role in modulating Maillard reaction kinetics during drying. While the impact of electric fields on nutritional compounds such as vitamins and antioxidants has been well documented, the role of waveform characteristics has been overlooked in the existing literature. Most of the studies focused on a single waveform and its effect at different intensities [3,4]. However, waveform can play a critical role in determining the efficiency of electroporation mechanism. For example, square-shaped waveforms have demonstrated superior energy efficiency and microbial inactivation capacity compared to exponential and sinusoidal pulses [5]. Nevertheless, designing a square waveform-producing system is more complex and challenging due to more requirements as pulse forming network, capacitors and inductors [6].

This study distinguishes itself from prior research by investigating some gaps as how moderate electric field strength, waveform type, and pulsing mode influence the retention of nutritional and chemical compounds in dried apple slices. It is focused on low electric field strength application to obtain optimum results with minimum energy requirement. Bazhal and Vorobiev stated that moderate electric field applications can be adequate to trigger electroplasmolysis below 0.3 kV/cm electric field strengths [7]. The effect of electroplasmolysis on some nutritional compounds such as ascorbic acid and β-carotene, as well as on the process contaminant HMF, was the primary focus. Ascorbic acid is vital for the nutritional and antioxidant profile of apples and is highly sensitive to thermal and oxidative degradation [8]. Similarly, β-carotene, a lipophilic provitamin A carotenoid, plays a dual role in health by mitigating oxidative stress. HMF, on the other hand, forms under heat in the presence of reducing sugars and amino acids, making its control critical for food safety and quality. HMF formation might be affected by processing temperature, water activity, sugar content, and amino acids [9].

This study evaluates the impact of electric field pretreatment on the retention of vitamin C, β-carotene, and HMF in dried apple slices, with a focus on optimizing electric field parameters (strength, waveform, and pulsing). A low electric field strength is aimed within 0.1–0.2 kV/cm in order to keep the voltage gradient low and present the advantages and disadvantages of electric field applications with lower energy consumption. By correlating electric field induced cellular changes with nutrient stability and Maillard reaction dynamics, this work aims to optimize drying efficiency by preserving nutritional quality.

## 2. Materials and Methods

### 2.1. Raw Material and Preparation

Apples of Malus domestica, Fuji genus, were obtained from a local market in Turkey and stored at 4 °C prior to experiments. The weight of the apples averaged between 150 g and 200 g. Fruits were washed and sliced to a thickness of 3 mm using a slicer. The slices were treated with electric field after slicing. All tests were performed in triplicate.

### 2.2. Electric Field Treatment and Drying

The electric field application was performed using a custom-built setup consisting of two parallel plate electrodes with a surface area of 150 cm^2^. The electrodes were spaced 10 mm apart, and the applied voltages were 100 V, 150 V, and 200 V. Both sinusoidal and square waveforms were generated using an AC power supply, with a frequency of 50 Hz and were applied continuously or with a pulse duration of 100 ms. The MEF treatment consisted of 60 pulses, resulting in a total active exposure time of 6 s. The overall treatment period was fixed at 1 min to allow for consistent comparison with continuous electric field applications and to remain within equipment limitations. The choice of treatment duration was informed by preliminary experiments, which indicated that longer total application times negatively impacted physical quality, particularly texture, in line with findings reported in previous studies [10]. The electric field strength (E) was calculated using the formula E = V/d, where V is the applied voltage and d is the distance between the electrodes. The resulting electric field strengths were 0.1, 0.15, and 0.2 kV/cm for increasing voltage levels, respectively.

**Figure 1:**
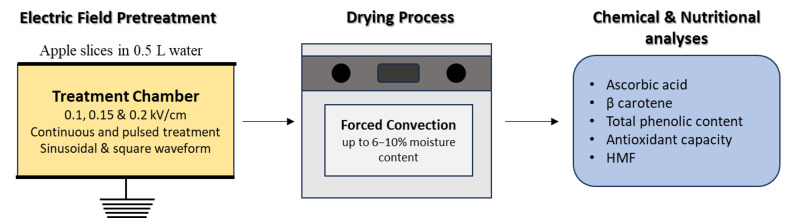
Electric field treatment conditions, processing parameters, and performed analyses.

Sliced apples were placed inside the electric field treatment chamber with a uniform thickness of 3 mm and placed between the electrodes. The chamber was filled with 0.5 L tap water at room temperature (Figure 1). The MEF treatment was applied for 1 min treatment time. Control experiments were conducted using untreated samples. After treatment, apple slices were drained quickly using tissue paper to remove excess water on the surface and dried to 6–10% moisture content using a convection oven at 75 °C. Drying was performed in a forced convection hot air oven at a temperature of 75 °C with an air velocity of approximately 1.2 m/s, a commonly used condition in apple drying and other fruit dehydration studies [2]. This temperature was selected as a compromise between achieving efficient drying rates and preserving nutritional quality, since lower temperatures would require prolonged drying times, potentially leading to oxidative degradation, while higher temperatures (>80 °C) could cause excessive loss of heat-sensitive nutrients such as vitamin C and β-carotene. Approximately 350 g of apple slices were placed on each tray in a single layer. The samples were dried until a target moisture content of 6–10% was reached, which typically required 3.5 to 4 h of drying time. The target final moisture content was set below 10%, in line with food safety standards, as moisture content below 10% combined with water activity below approximately 0.60–0.65 is known to inhibit microbial growth and ensure shelf stability [11]. Maintaining low final moisture content is also consistent with previous PEF-assisted drying studies, such as Chauhan et al. (2018), who reported target moisture levels of 6–7% for dried apple slices [12].

### 2.3. Moisture Content

The moisture content of the apple samples was determined using a gravimetric method. The samples were first ground to ensure homogeneity and then dried in a convection oven (Memmert GmbH + Co. KG, UNE 400, Schwabach, Germany) at a controlled temperature of 105 °C until a constant weight was achieved, indicating the completion of moisture removal [13].

### 2.4. Determination of Ascorbic Acid (Vitamin C) Content

Ascorbic acid quantification was conducted under cold, light-protected conditions to prevent oxidation. Samples were homogenized in a pre-chilled grinder submerged in an ice-water bath. After homogenization, samples were immediately stored on ice in aluminum foil-wrapped containers. A 5 g aliquot was weighed and mixed with 6% (*w*/*v*) meta-phosphoric acid solution of 50 mL. The mixture was blended for 1 min in an ice-cooled, foil-covered glass beaker using a hand blender, followed by 20-min sonication in an ultrasonic bath (ice-cooled, light-protected). The extract was filtered through qualitative filter paper and a 0.45 μm PTFE/nylon membrane into amber vials to minimize light exposure.

Chromatographic separation using High Performance Liquid Chromatography (HPLC) (LC-2050C, Shimadzu Corp, Kyoto, Japan) employed a GL Sciences Inertsil ODS-4 column (5 μm, 4.6 mm × 250 mm) at 20 °C, with a mobile phase flow rate of 0.6 mL/min. Ascorbic acid was detected at 244 nm using a photodiode array (PDA) detector. Prior to analysis, the column was conditioned for 30 min to stabilize the baseline (±1 mAU). A 20 μL injection volume was used for each run. All extractions and analyses were performed in triplicate [14].

### 2.5. Determination of β-Carotene Content

The procedure to assess β-carotene content is adapted from Barba et al. with modifications [15]. For β-carotene quantification, samples were homogenized under cold conditions to minimize degradation using a blender (GM 200, Retsch GmbH, Haan, Germany). Briefly, pre-cooled samples were ground in an ice-cooled blender until complete homogenization was achieved. Homogenized samples were immediately stored on ice in aluminum foil-wrapped containers to prevent light exposure. A 1.25 g aliquot of the homogenate was weighed and dissolved in 100% methanol. The mixture was sonicated at maximum power in an ultrasonic water bath (Memmert GmbH + Co. KG, Schwabach, Germany) for 45 min to facilitate extraction. Subsequently, the solution was centrifuged at 3000 rpm for 30 min (4 °C) (3-16KL, Sigma Laborzentrifugen GmbH, Harz, Germany), and the supernatant was collected. The extraction process was repeated until the residue became colorless, ensuring exhaustive β-carotene recovery. Combined supernatants were filtered through a 0.45 μm membrane filter and transferred to amber vials for HPLC analysis.

HPLC analysis was performed using a GL Sciences Inertsil ODS-3 column (10 μm, 3.9 mm × 300 mm) maintained at 40 °C. The mobile phase flowed at 1 mL/min, and β-carotene was detected at 475 nm using a photodiode array (PDA) detector. A 20 μL aliquot was injected for each run. All procedures were conducted in triplicate to ensure analytical reproducibility.

### 2.6. Total Phenolic Content Determination

The total phenolic content (TPC) of fruit samples was determined using the Folin–Ciocalteu method. First, 5 g of the sample was homogenized in 50 mL of methanol using a blender and subjected to extraction in a water bath at 40 °C for 60 min. A total of 50 µL of the sample extract was mixed with 3 mL of distilled water, 250 µL of Folin–Ciocalteu phenol reagent, and 750 µL of 7% K_2_CO_3_ solution. The mixture was vortexed and allowed to react for 8 min at room temperature. Subsequently, 950 µL of distilled water was added, and the solution was incubated in the dark at room temperature for 2 h. Absorbance was measured at 750 nm using a UV-Vis spectrophotometer (Lambda 35, Perkin Elmer, Springfield, IL, USA), with a blank solution for baseline correction. The results were expressed as gallic acid equivalents (µg GAE/100 g sample) [16].

### 2.7. Antioxidant Capacity

The antioxidant capacity of the apple samples was determined using the DPPH radical scavenging assay. The assay is adapted from Matys et al. with slight modifications. 2.5 g of the homogenized sample was mixed with 10 mL of 80% methanol solution and further homogenized [17]. The mixture was vortexed and centrifuged at 4000 rpm for 10 min. The supernatant was then filtered through Whatman No. 1 filter paper (Cytiva, Wilmington, DE, USA). A freshly prepared 0.1 mM DPPH solution was used to assess antioxidant activity. In a test tube, 200 μL of the extract was mixed with 4 mL of DPPH solution and incubated in the dark at room temperature for 20 min. A blank sample containing only 80% methanol and DPPH solution was prepared under the same conditions. Absorbance values were measured at 517 nm using a UV-Vis spectrophotometer.

### 2.8. HMF Determination

For HMF quantification, homogenized samples were prepared using a blade grinder. A 10 g aliquot of the homogenate was weighed, mixed with 25 mL of ultrapure water, and transferred to a 50 mL volumetric flask. The mixture was stirred for 15 min to extract HMF. For clarification, 2 g of the sample was treated with 4 mL each of Carrez I (15 g K_4_ [Fe(CN)_6_]·3H_2_O in 100 mL ultrapure water) and Carrez II (30 g ZnSO_4_·7H_2_O in 100 mL ultrapure water) to precipitate interfering substances. The solution was diluted to 100 mL with deionized water, stirred for 30 min, and filtered through a 0.45 μm syringe filter into amber vials to prevent light degradation.

HPLC analysis was performed using a methanol:water mobile phase (10:90, *v*/*v*) at a flow rate of 1 mL/min. A 20 μL injection volume was introduced into the HPLC system equipped with a column oven maintained at 25 °C. HMF was detected at 284 nm using a diode array detector (DAD), with a retention time of 11.140 min. All analyses were conducted in triplicate to ensure precision [18].

### 2.9. Statistical Analysis

All experimental data were statistically analyzed using Minitab 21.1 Statistical Software for Windows^®^ (Minitab Inc., State College, PA, USA). Analysis of variance (ANOVA) was performed to assess the significance of the effects of MEF application on the dried apple samples.

## 3. Results

The effects of MEF pretreatment and hot-air drying on key nutritional and quality parameters of apple slices were systematically evaluated. Table 1 summarizes the measured values of vitamin C, β-carotene, antioxidant activity (DPPH), HMF, and total phenolic content (TPC) across different treatment conditions, including fresh samples, samples dried without MEF, and samples treated with MEF at varying field strengths prior to drying. Detailed analyses and discussions for each parameter are presented in the following sections.

### 3.1. Effect of Electric Field on Vitamin C Content of Apples

**Figure 2:**
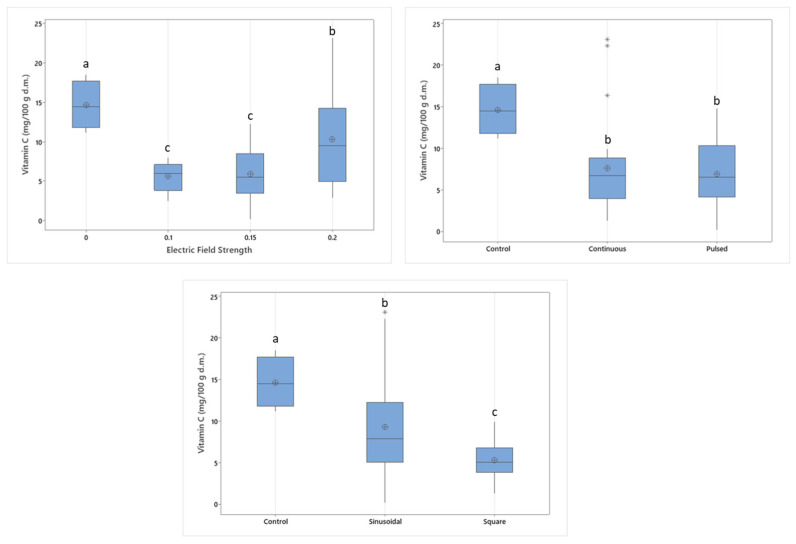
The effect of electric field application parameters, individually, on the ascorbic acid content of dried apples. Different letters on a single graph indicate significant differences (*p* < 0.05). * Data points marked with an asterisk represent statistical outliers. Mean values are represented by circles.

The change in the vitamin C content of samples due to varying electric field strengths is given in Figure 2. Electric field application significantly affected the vitamin C content. Untreated control samples have the highest vitamin C content, and the minimum vitamin C content is obtained at 0.1 kV/cm pretreatment. A significant difference between control and MEF-treated samples was also observed in different studies. Ciurzynska et al. reported a dramatic decrease between control and MEF-treated samples at varying drying temperatures of 60, 70, and 80 °C but the decrease correlated with increasing electric field strengths [19].

The reason for obtaining higher vitamin C content at 0.2 kV/cm electric field treatment might be the result of faster inactivation of enzymes at higher intensities. Electric field strength has a critical effect on the thermal stability of ascorbic acid oxidase, as the electric field strength increases, Ea values decrease, and k values increase, meaning that the enzyme becomes more thermolabile [20]. Leong and Oey explained that this situation might occur due to intracellular changes caused by the permeability of MEF signals in food tissue, making ascorbic acid oxidase more fragile against thermal effects. As the effectiveness of the enzyme decreases, a higher amount of vitamin C can be retained [20].

The vitamin C contents of continuous application and pulsed application of these electric field strengths did not show a significant difference. Applying electric field in pulses generally showed an increase in the drying rate due to disruptions of food cells, which leads to faster removal of moisture from the food. Lamanauskas et al. applied PEF on Actinidia kolomikta, a fruit remarkably high in vitamin C content, with 5 kV/cm electric field and pulses of 20 μs duration at 20 Hz [21]. The amount of ascorbic acid in control samples and treated samples did not show any difference. The application of PEF pretreatment on drying of A. kolomikta is considered useful to increase drying efficiency of the fruits without causing any change in the ascorbic acid content [21]. In another study conducted by Yu, Jin, and Xiao (2017) on blueberries, which have similar ascorbic acid levels, PEF pretreatment on hot air drying did not affect the vitamin C content [22]. However, the pretreatment prior to vacuum drying showed good vitamin C retention at 75 °C as 45.5% [22]. Zhang et al. (2015) investigated the effect of PEF on vitamin C structure and its antioxidative properties and stated that PEF treatment can cause modifications in the molecular structure of vitamin C by changing its conformation from enol to keto form [23]. The potential of PEF treatment to enhance the antioxidant properties of vitamin C without damage is also demonstrated in DPPH radical scavenging and reducing power tests [23].

The application of PEF with different waveforms on apple drying was also investigated. Vitamin C is retained significantly better in sinusoidal wave application compared to square-shaped waves, while control samples had the highest vitamin C content. Square waves are known for their higher efficiency compared to other waveforms and more detrimental cell disintegration levels [5]. This detrimental effect seems to also be effective in degrading vitamin C content. Square wave profiles can also change the electrical conductivity of the food components. As conductivity increases, the electroporation mechanism might slow down and the drying rate might be lowered. Since the change in the rate of moisture removal can affect temperature increase, vitamin C degradation might be diminished indirectly.

**Figure 3:**
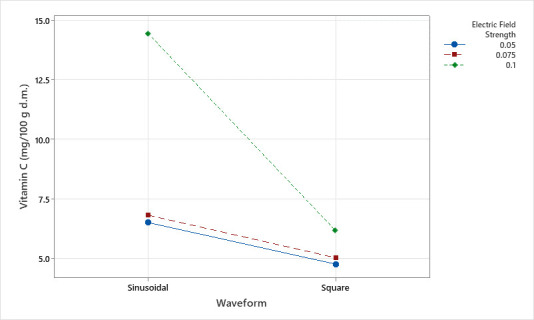
Two-way interaction of electric field strength and waveform on vitamin C content.

The two-way interaction of electric field strength and waveform also had a significant influence on the vitamin C content (*p* < 0.05). When the electric field is applied in sine form, the intensity of the electric field affects the vitamin C content. Although the effect of square waveforms also increases with higher electrical field strengths, square waves are more disruptive to vitamin C molecules, as can be seen in Figure 3. Square waveforms are considered more impactful and more energy efficient because the voltage duration of peaks is longer, besides their complexity of system design [6].

### 3.2. The Effect of Electric Application on β-Carotene Content of Dried Apples

**Figure 4:**
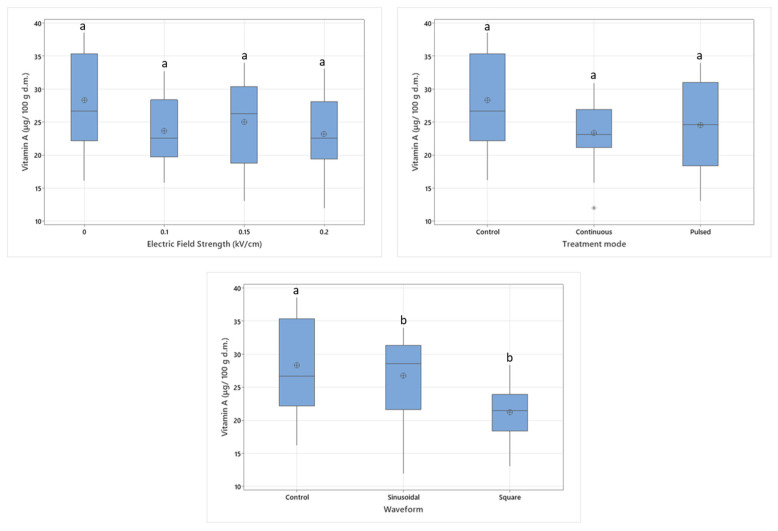
The effect of individual electric field application parameters on the β-carotene content of dried apples. Different letters on a single graph indicate significant differences (*p* < 0.05). * Data points marked with an asterisk represent statistical outliers. Mean values are represented by circles.

Apples are known as a good source of carotenoids that possess beneficial health effects for their antioxidant properties and their impact on color and sensory attributes [24]. In this study, apples subjected to varying electric field strengths prior to hot-air drying did not differ in β-carotene content (*p* > 0.05). Even though the electric field strength was found to be ineffective, untreated samples were found to have a higher amount of β-carotene (Figure 4). The amount of β-carotene obtained in control samples of Fuji apples is in agreement with the amounts stated by Felicetti and Schrader [25] and Jia et al. [26].

Huang et al. observed no significant difference in β-carotene content in apricot samples treated with 0.625 and 1.25 kV/cm PEF (*p* > 0.05) [27]. However, it was reported that β-carotene content increased compared to fresh samples, which might be caused by the higher extraction yield of β-carotene due to improved electroporation of fruit cells [27]. β-carotene is a provitamin A carotenoid, which can be converted by the human body into vitamin A. Sánchez-Moreno et al. [28] reported that the vitamin A content of orange juice was not affected significantly by 35 kV/cm PEF treatment. Evrendilek et al. [29] observed no difference with 30 kV/cm PEF treatment on apricot nectar [28,29]. In another study by Kim et al. [30], the total carotenoid content was found to be retained significantly better in dried carrot samples (*p* < 0.05).

Similarly, to the effect of electric field strength, continuous and pulsed electric field treatments did not show a significant difference between each other or compared to the control samples. The retention of carotenoids might also be higher due to partial inactivation of degradation enzymes [31]. Furthermore, the insolubility of carotenoids in water may limit their release in juice and may lead to better retention in pomace. López-Gámez et al. [32] stated that the release of carotenoids from pomace to juice is not efficient at high electric field strengths and frequencies above 10 Hz.

Despite the fact that β-carotene content remained unaffected by the strength and pulse of electric field application, the waveform of the electric field had a significant influence (*p* < 0.05). Control samples, sinusoidal and square wave apples contained 28.36, 26.74, and 21.21 μg/ 100 g d.m. β-carotene, respectively. The highest β-carotene content was obtained in control samples and was statistically not different from sinusoidal electric field application. However, square waves diminished β-carotene content significantly, regardless of the stable nature of β-carotene against electric field application. This could be the result of an extra detrimental effect of square waves due to their longer peak voltage duration [6]. Although the short pulse duration (60 ms) suggests minimal overall heating, localized heating from high field gradients may have contributed to β-carotene loss and warrants further investigation.

### 3.3. The Effect of Electric Field Application on the Antioxidant Capacity

**Figure 5:**
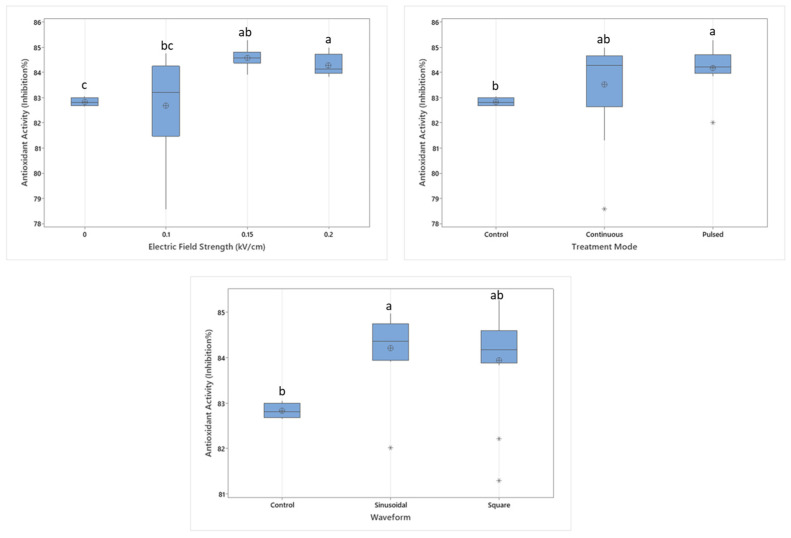
The effect of individual electric field application parameters on the antioxidant activity of dried apples. Different letters on a single graph indicate significant differences (*p* < 0.05). * Data points marked with an asterisk represent statistical outliers. Mean values are represented by circles.

The effects of MEF parameters, including field strength, pulse treatment, and waveform, on the antioxidant capacity of dried apples were investigated (Figure 5). The findings indicate that increasing the field strength from 0.1 to 0.15 kV/cm resulted in a significant enhancement of DPPH radical scavenging activity (*p* < 0.05). However, a decline was observed at 0.2 kV/cm, suggesting that higher electric field strengths may lead to oxidative degradation of samples. Similarly, Zhang et al. [23] reported that PEF-treated samples exhibited higher antioxidant capacities compared to untreated control samples. Moreover, increasing the exposure time of PEF treatment did not lead to a reduction in DPPH radical scavenging activity; instead, an increase was observed, which may be attributed to changes in molecular structure [23]. Similarly, Shiekh et al. [33] reported that untreated control samples of custard apples had lower antioxidant activity compared to PEF-treated samples. In another study, PEF application showed a negative impact on the antioxidant activity; however, as the energy level increased, the antioxidant capacity of the samples showed a slight increase [17].

Similarly, a slight increase in antioxidant activity was observed with an increase in pulse treatment; however, results imply that the effect is not statistically significant (*p* > 0.05). The waveform effect on the antioxidant activity is found to be likely negligible since the difference between sinusoidal and square waveforms was minimal, with the sinusoidal waveform exhibiting a marginally higher antioxidant activity. Santos et al. [34] reported that the PEF application may increase the antioxidant activity in potato samples compared to control. Also it was stated that as the number of pulses increases, the antioxidant activity increases even higher.

**Figure 6:**
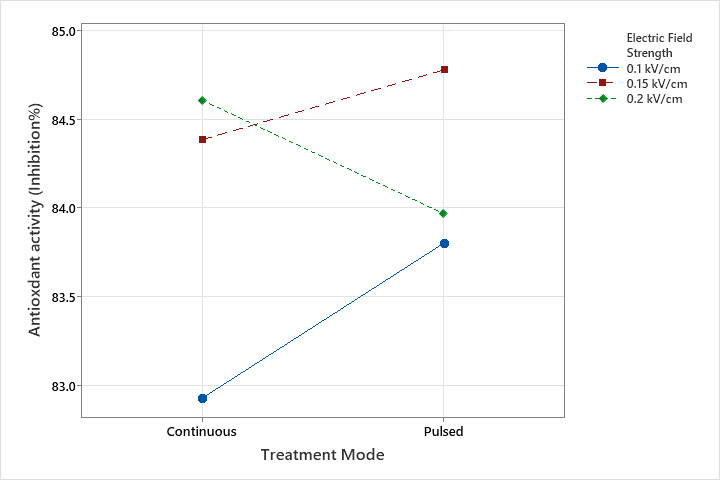
The interaction graph of electric field strength and treatment mode on the antioxidant activity.

Figure 6 illustrates the interaction between treatment mode and electric field strength on antioxidant activity. The results indicate that at lower electric field strengths (0.1 and 0.15 kV/cm), pulsed treatment led to a noticeable increase in antioxidant activity compared to continuous treatment. However, at 0.2 kV/cm, pulsed treatment resulted in a slight decrease in antioxidant activity. These findings suggest that while increasing electric field strength and applying pulse treatment can enhance antioxidant activity, their combined application may, in some cases, lead to lower antioxidant levels. This aligns with previous studies indicating that moderate PEF intensities can enhance the activity of bioactive compounds, whereas excessive or suboptimal parameter combinations may diminish these benefits [17,33,34].

### 3.4. The Effect of Electric Field Application on Total Phenolic Content

TPC of dried apples was also significantly affected by electric field treatment conditions. Untreated samples had higher TPC, while increased levels of electric field strength elevated the retention of phenolic compounds, resulting in lower TPC values. Electric field application within the 0.1–0.2 kV/cm range did not show a significant difference (*p* > 0.05).

Higher treatment intensity improves electroporation efficiency, leading to greater extraction yields. Although thermal exposure was lower in treated apple slices due to faster drying, the formation of micropores increased leakage of phenolic compounds, facilitating their release from the fruit tissue [33]. A similar effect was reported by Matys et al. [17], where increased permeability due to electroporation led to lower phenolic content and some phenolic degradation caused by thermal exposure. Although there is a reduction in antioxidant activity, the TPC values did not show a corresponding decline, suggesting that other mechanisms may be involved, such as the composition and reactivity of phenolic compounds and degradation of non-phenolic antioxidants.

**Figure 7:**
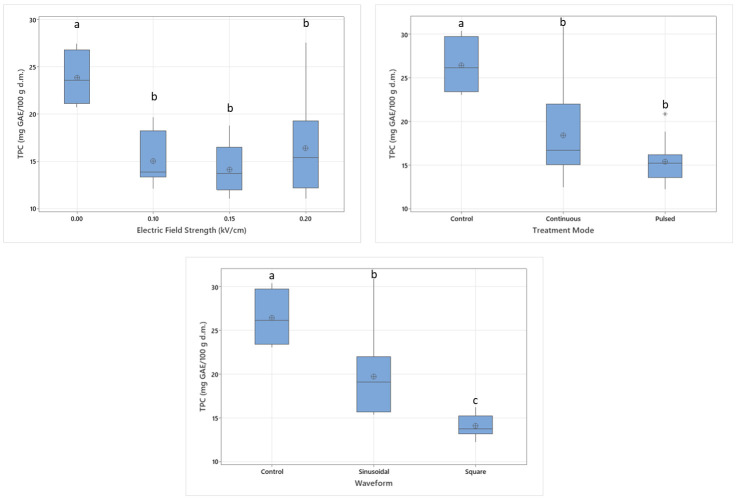
The effect of individual electric field application parameters on TPC of dried apples. Different letters on a single graph indicate significant differences (*p* < 0.05). * Data points marked with an asterisk represent statistical outliers. Mean values are represented by circles.

Square waves had a more detrimental effect on phenolic compounds, lowering TPC compared to both sinusoidal wave-applied samples and the control, similar to their impact on vitamin C content and antioxidant activity (*p* < 0.05). Although electric field treatment resulted in lower TPC than untreated samples, the effect of treatment mode was not found to be significant (*p* > 0.05) (Figure 7). Roshanak et al. [35] stated the possibility of an increase in TPC after drying due to the inactivation of enzymes that interact with phenolics in fresh fruit. Additionally, longer drying times and the associated stress factors may induce the formation of new phenolic compounds as a protective response in fruit samples.

### 3.5. The Effect of Electric Field Application on the HMF Content of Dried Apples

**Figure 8:**
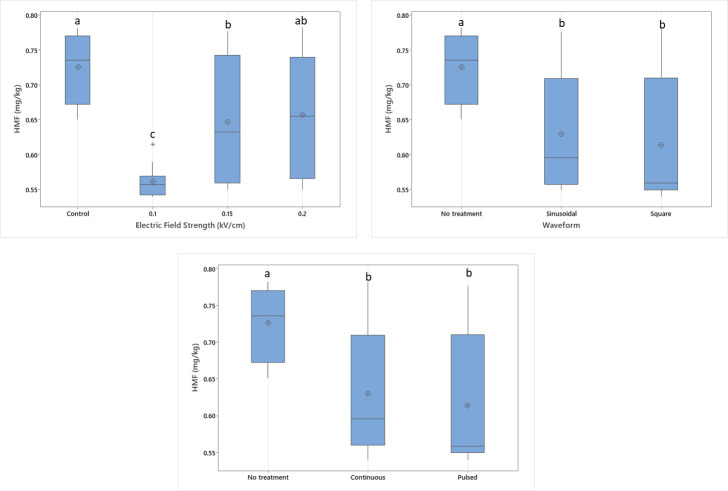
The effect of individual electric field application parameters on the HMF content of dried apples. Different letters on a single graph indicate significant differences (*p* < 0.05). * Data points marked with an asterisk represent statistical outliers. Mean values are represented by circles.

HMF content is not expected to be present in fresh foods, and its content increases with thermal treatment [36]. The highest HMF content was found in control samples as 0.73 mg/kg and the lowest HMF content was in samples treated with 0.1 kV/cm voltage (Figure 8). Electric field application has a lowering effect on HMF content; however, increasing the electric field strength by increasing the voltage did not have any effect on HMF formation. Both pulsed and continuous electric field applications resulted in similar HMF levels, which were lower than those observed in untreated control samples. The inhibiting effect of MEF on HMF formation may be attributed to faster drying resulting from an improved drying rate. However, intermediate moisture measurements were not performed in this study; therefore, drying rate assessments are based only on overall drying time and final moisture content. Future research should include detailed drying curves to better characterize drying kinetics and to clarify the effect of drying rate on HMF content. The electric field waveform did not influence the HMF content; sinusoidal and square wave applications showed similar HMF levels of 0.63 and 0.61 mg/kg, respectively, which are lower than those of the control samples.

Low levels of HMF content are probably due to faster drying of samples compared to control samples. Nonetheless, the reason for obtaining the lowest HMF content at lower electric field application might be due to water release limitation. Stronger electric field applications might increase electroporation on fruit cells and improve the rate of mass transfer of water. Since the removal of water lowers the water activity, Maillard reactions are affected and slowed down. By slowing down the HMF formation mechanism, the amount of HMF formed might be lowered. The HMF content of dried apple samples is similar to that reported by Wojdyło et al. [9]. The effect of electric fields on HMF content is generally investigated in fruit juice samples due to limitations on maximum HMF content. Makroo, Srivastava, and Jabeen [37] applied mild electric field ohmic heating to pineapple juice and obtained an increase in the HMF content with increasing voltage gradient. They stated that this increase might be caused by electric fields triggering non-enzymatic reactions and leading to more HMF formation [37]. In another study conducted on sour cherry juice, PEF treatment showed no significant effect on furfural and HMF content (*p* > 0.05) [38]. Similarly, PEF treatment showed no altering effect on HMF formation in apple juice samples (*p* > 0.05) [39].

## 4. Conclusions

Electric field treatment significantly influences the nutritional quality of dried apple slices, with pulse application and waveform selection playing a significant role. Unlike high-intensity PEF treatments that often induce irreversible electroporation and severe tissue disruption, the moderate electric field applied in this study likely resulted in reversible electroporation and minor alterations of cell membrane permeability. This mechanism allows for enhanced mass transfer during drying while better preserving tissue structure and nutritional quality.

Sinusoidal waveforms at 0.2 kV/cm optimally preserved vitamin C and minimized β-carotene degradation, whereas square waves, despite their energy efficiency, accelerated nutrient losses due to harsher electroporation. The moderate electric field parameters used in this study are well within the ranges reported in the literature as safe and non-destructive for food applications [2]. These parameters are not expected to induce significant chemical modifications or the formation of harmful compounds. However, future work should include comprehensive safety assessments to further confirm the applicability of MEF treatments in food processing.

HMF formation was reduced in electric-field-treated samples, attributed to faster drying and shortened thermal exposure. However, the trade-off between enhanced drying efficiency and nutrient retention necessitates parameter-focused optimization. For instance, sinusoidal PEF at moderate field strengths (0.15–0.2 kV/cm) balances vitamin C retention and HMF reduction, while square waves may be reserved for applications prioritizing drying speed over nutrient conservation. Electric field treatment increased the antioxidant activity of the dried apples; however, higher electric field strengths and some treatment combinations may lead to reduced antioxidant activity levels. Similarly, electric field strengths and pulse treatments increased the permeability of food matrices and leading to the liberation of phenolic compounds and decreased TPC levels. Future studies should explore hybrid waveforms and parameter optimization to find suitable application procedures for diverse fruit matrices. Future research should incorporate correlation analysis and multivariate techniques to elucidate the relationships among nutritional and antioxidant parameters in MEF-treated apple slices. Additionally, future studies should include comprehensive evaluations of color, texture, and sensory properties to complement the nutritional data and to support the optimization of processing conditions for apple products.

## Figures and Tables

**Table 1:** Changes in the nutritional and antioxidant parameters (vitamin C, β-carotene, antioxidant activity, and TPC) of apple slices at different processing stages: fresh untreated, after MEF treatment (before drying), and after MEF + drying.

Wave Type	Treatment Mode	Treatment Condition	Vitamin C (mg/100 g d.m.)	β-Carotene (mg/100 g d.m.)	Antioxidant Activity (DPPH)	TPC (mg GAE /100 g d.m.)	HMF (mg/kg d.m.)
-	-	Before drying	89.16 ± 14.40	60.86 ± 22.12	70.21 ± 8.79	142.81 ± 4.86	0.73 ± 0.05
-	-	After drying, no MEF	14.66 ± 3.08 ^ab^	28.36 ± 7.03 ^ab^	82.83 ± 0.17 ^bc^	26.45 ± 1.64 ^a^	0.73 ± 0.05 ^a^
Sinus wave	Continuous	0.1 kV/cm MEF	7.51 ± 0.45 ^cd^	21.65 ± 4.45 ^abcde^	84.1 ± 0.15 ^ab^	21.71 ± 3.92 ^ab^	0.59 ± 0.02 ^b^
	Continuous	0.15 kV/cm MEF	5.77 ± 3.23 ^cd^	28.80 ± 1.63 ^abcd^	84.29 ± 0.53 ^ab^	18.08 ± 1.06 ^abc^	0.71 ± 0.00 ^a^
	Continuous	0.2 kV/cm MEF	16.39 ± 8.98 ^a^	17.41 ± 3.93 ^de^	84.75 ± 0.25 ^a^	16.05 ± 0.00 ^abc^	0.60 ± 0.01 ^b^
	Pulsed	0.1 kV/cm MEF	5.52 ± 1.62 ^cd^	30.74 ± 1.69 ^abc^	83.14 ± 1.59 ^abc^	14.24 ± 0.27 ^bc^	0.56 ± 0.00 ^b^
	Pulsed	0.15 kV/cm MEF	7.87 ± 5.42 ^bcd^	32.41 ± 2.47 ^a^	84.66 ± 0.27 ^a^	13.92 ± 0.25 ^c^	0.76 ± 0.01 ^a^
	Pulsed	0.2 kV/cm MEF	12.55 ± 1.68 ^abc^	29.43 ± 2.62 ^abc^	83.94 ± 0 ^abc^	17.14 ± 0.00 ^abc^	0.55 ± 0.00 ^b^
Square wave	Continuous	0.1 kV/cm MEF	4.94 ± 1.46 ^cd^	22.67 ± 1.40 ^abcde^	81.76 ± 0.66 ^c^	13.12 ± 0.89 ^c^	0.55 ± 0.02 ^b^
	Continuous	0.15 kV/cm MEF	3.88 ± 2.25 ^d^	23.00 ± 1.75 ^abcde^	84.48 ± 0.21 ^ab^	12.93 ± 1.12 ^c^	0.56 ± 0.01 ^b^
	Continuous	0.2 kV/cm MEF	7.43 ± 2.50 ^cd^	26.70 ± 1.76 ^abcde^	84.18 ± 0 ^abc^	12.11 ± 0.01 ^c^	0.77 ± 0.02 ^a^
	Pulsed	0.1 kV/cm MEF	4.58 ± 1.79 ^cd^	19.61 ± 1.75 ^bcde^	84.46 ± 0.4 ^ab^	12.71 ± 0.83 ^c^	0.54 ± 0.01 ^b^
	Pulsed	0.15 kV/cm MEF	6.17 ± 2.28 ^cd^	15.90 ± 2.32 ^e^	84.89 ± 0.54 ^a^	11.53 ± 0.64 ^c^	0.56 ± 0.01 ^b^
	Pulsed	0.2 kV/cm MEF	4.91 ± 1.73 ^cd^	19.36 ± 1.74 ^cde^	83.98 ± 0.13 ^ab^	12.79 ± 1.84 ^c^	0.71 ± 0.01 ^a^

Data are presented as mean ± standard deviation (n = 3). Different letters in the same column indicate significant differences (*p* < 0.05).

## Data Availability

The original contributions presented in this study are included in the article. Further inquiries can be directed to the corresponding author.

## Abbreviations

The following abbreviations are used in this manuscript:

MEF	Moderate electric field
PEF	Pulsed electric field
HMF	Hydroxymethylfurfural
V	Voltage
AC	Alternating current
Hz	Hertz
d	Distance
HPLC	High performance liquid chromatography
PTFE	Polytetrafluoroethylene
PDA	Photo diode array
DAD	Diorde array detector
TPC	Total phenolic content
UV-Vis	Ultraviolet–visible
GAE	Gallic acid equivalent
DPPH	2,2-diphenyl-1-picrylhydrazyl.
